# DOCK11 promotes HBV cccDNA formation through a PARP1-dependent mechanism

**DOI:** 10.1128/spectrum.04140-25

**Published:** 2026-06-16

**Authors:** Hideo Takayama, Kouki Nio, Kazuyuki Kuroki, Ying-Yi Li, Saiho Sugimoto, Tetsuro Shimakami, Kazuhisa Murai, Kazunori Kawaguchi, Takumi Nishiuchi, Shuichi Kaneko, Masao Honda, Taro Yamashita

**Affiliations:** 1 Department of Gastroenterology, Graduate School of Medical Sciences, Kanazawa University 12858 , Kanazawa, Japan; 2 Department of Clinical Laboratory Medicine, Graduate School of Medical Sciences, Kanazawa University 12858 , Kanazawa, Japan; 3 Division of Life Science, Graduate School of Natural Science and Technology, Kanazawa University 300662, Kanazawa, Japan; Institute for Biomedical Research on Retroviruses and AIDS (INBIRS), Buenos Aires, Argentina

**Keywords:** hepatitis B virus, cccDNA, rcDNA, cccDNA formation, DOCK11, PARP1, DNA repair

## Abstract

Hepatitis B virus (HBV) infection remains a major global health concern due to the persistence of covalently closed circular DNA (cccDNA) in the hepatocyte nucleus, which contributes to viral persistence and hepatocarcinogenesis despite antiviral therapy. Poly(ADP-ribose) polymerase 1 (PARP1) has been reported to bind to HBV relaxed circular DNA (rcDNA) and contribute to the conversion of rcDNA to cccDNA. We previously identified dedicator of cytokinesis 11 (DOCK11) as a host factor whose suppression reduced cccDNA levels in HBV infection models; however, the precise role of DOCK11 in the process of cccDNA formation remains unclear. In this study, we investigated the role of DOCK11 in HBV cccDNA formation and the underlying mechanisms. Using a minicircle HBV cccDNA reporter, we observed that luciferase activity declined significantly in DOCK11-depleted hepatoma cells over time, consistent with impaired cccDNA maintenance. In a cell-free assay using rcDNA and nuclear extracts, DOCK11 overexpression significantly enhanced cccDNA formation, while DOCK11 knockout significantly suppressed it. Co-immunoprecipitation suggested that DOCK11 forms a multiprotein complex with HBV DNA. Furthermore, liquid chromatography-mass spectrometry analysis identified PARP1 as a significant component of this complex. Notably, while DOCK11 did not affect PARP1 expression or enzymatic activity, DOCK11 knockout significantly reduced PARP1 recruitment to UV-irradiated DNA damage sites and its association with rcDNA, suggesting a supportive role for DOCK11 in PARP1 recruitment to rcDNA. In summary, DOCK11 promotes HBV cccDNA formation, possibly through its interaction with HBV DNA and PARP1. These findings position DOCK11 as a promising therapeutic target for cccDNA reduction in chronic HBV infection.

**IMPORTANCE:**

Hepatitis B virus (HBV) causes chronic liver infection, affecting hundreds of millions of people worldwide and increasing the risk of cirrhosis and hepatocellular carcinoma. Persistent HBV infection is maintained by covalently closed circular DNA (cccDNA), which is resistant to current antiviral therapies. Understanding cccDNA maintenance mechanisms, including cccDNA formation, is therefore critical for developing curative treatments. Poly(ADP-ribose) polymerase 1 (PARP1) is known to bind HBV relaxed circular DNA (rcDNA) and contribute to the conversion of rcDNA to cccDNA. In this study, we identified the host protein dedicator of cytokinesis 11 as a host regulator of HBV cccDNA formation through its interaction with PARP1, an enzyme involved in DNA repair. Our findings reveal a previously unrecognized mechanism by which HBV exploits host cellular machinery to persist in hepatocytes. By inhibiting this pathway, our work highlights potential targets for novel therapies aimed at eliminating cccDNA and achieving a functional cure for HBV infection.

## INTRODUCTION

Hepatitis B virus (HBV) infection remains a major global health concern, with an estimated 254 million people chronically infected and approximately 1.2 million new infections occurring annually (1). The infection must be overcome to prevent life-threatening liver diseases such as cirrhosis and hepatocellular carcinoma.

HBV primarily exists as relaxed circular DNA (rcDNA) in viral particles. After HBV infection, HBV rcDNA is transported to the nucleus, where it is converted into HBV covalently closed circular DNA (cccDNA) using host cellular machinery. HBV cccDNA is characterized by its stable, fully closed circular structure. It persists in the nucleus and serves as the transcriptional template for most viral RNAs, playing an essential role in HBV replication. Although current nucleos(t)ide analogs used in clinical practice effectively inhibit reverse transcription of the viral genome, they cannot eliminate HBV cccDNA from the nucleus; therefore, complete HBV eradication is not currently achievable.

Several mechanisms involved in the maintenance of HBV cccDNA have been reported. One particularly important contributor is HBV cccDNA formation. *In vitro* biochemical studies have shown that a minimal set of host factors required for HBV cccDNA formation consists of lagging-strand DNA synthesis-related proteins, including proliferating cell nuclear antigen (PCNA), replication factor C (RFC), DNA polymerase δ (POLδ), flap endonuclease 1 (FEN1), and DNA ligase 1 (LIG1) (2, 3). During this process, FEN1 mediates the cleavage of flap structures on rcDNA (4), while LIG1 and LIG3 contribute to the ligation steps required for rcDNA-derived cccDNA formation (5). In addition to these minimal host factors, several auxiliary host proteins that facilitate or regulate distinct steps of cccDNA formation have been reported, including cellular DNA topoisomerases (6), DNA polymerase α involved in intracellular cccDNA amplification (7), the ataxia telangiectasia and Rad3-related (ATR)–checkpoint kinase 1 (CHK1) DNA damage response pathway required for rcDNA processing (8), DNA polymerase κ functioning during *de novo* HBV infection (9), and poly(ADP-ribose) polymerase 1 (PARP1) (10). As one such factor, PARP1, a DNA damage sensor, binds to HBV rcDNA and contributes to the conversion of rcDNA to cccDNA, potentially through interactions with other host DNA repair proteins such as RFC1, X-ray repair cross-complementing protein 1 (XRCC1), PCNA, and LIG3 (10).

Using single-cell transcriptome analysis, we previously identified dedicator of cytokinesis 11 (DOCK11) as a key host molecule involved in HBV maintenance (11). DOCK11 functions as a guanine nucleotide exchange factor for cell division cycle 42 (CDC42), thereby influencing cell motility and intracellular signaling. We have demonstrated that knockdown of DOCK11 using short hairpin RNA (shRNA) reduced HBV cccDNA levels in HBV-infected primary human hepatocytes isolated from humanized liver chimeric mice (11), and DOCK11 knockdown by small interfering RNA (siRNA) similarly reduced cccDNA levels in HBV-infected mice (12). Mechanistically, DOCK11 is thought to be involved in the retrograde trafficking of HBV (13), activation of the ATR signaling pathway in UV-mediated DNA damage (14), and regulation of the subnuclear localization of cccDNA to facilitate its association with histone H3 lysine 4 trimethylation and RNA polymerase II (15). However, the precise role of DOCK11 in the process of HBV cccDNA formation remains unclear. In this study, we investigated the role of DOCK11 in the regulation of HBV cccDNA formation and the underlying mechanisms using an *in vitro* cccDNA formation assay.

## RESULTS

### DOCK11 contributes to the maintenance of HBV cccDNA

**Fig 1:**
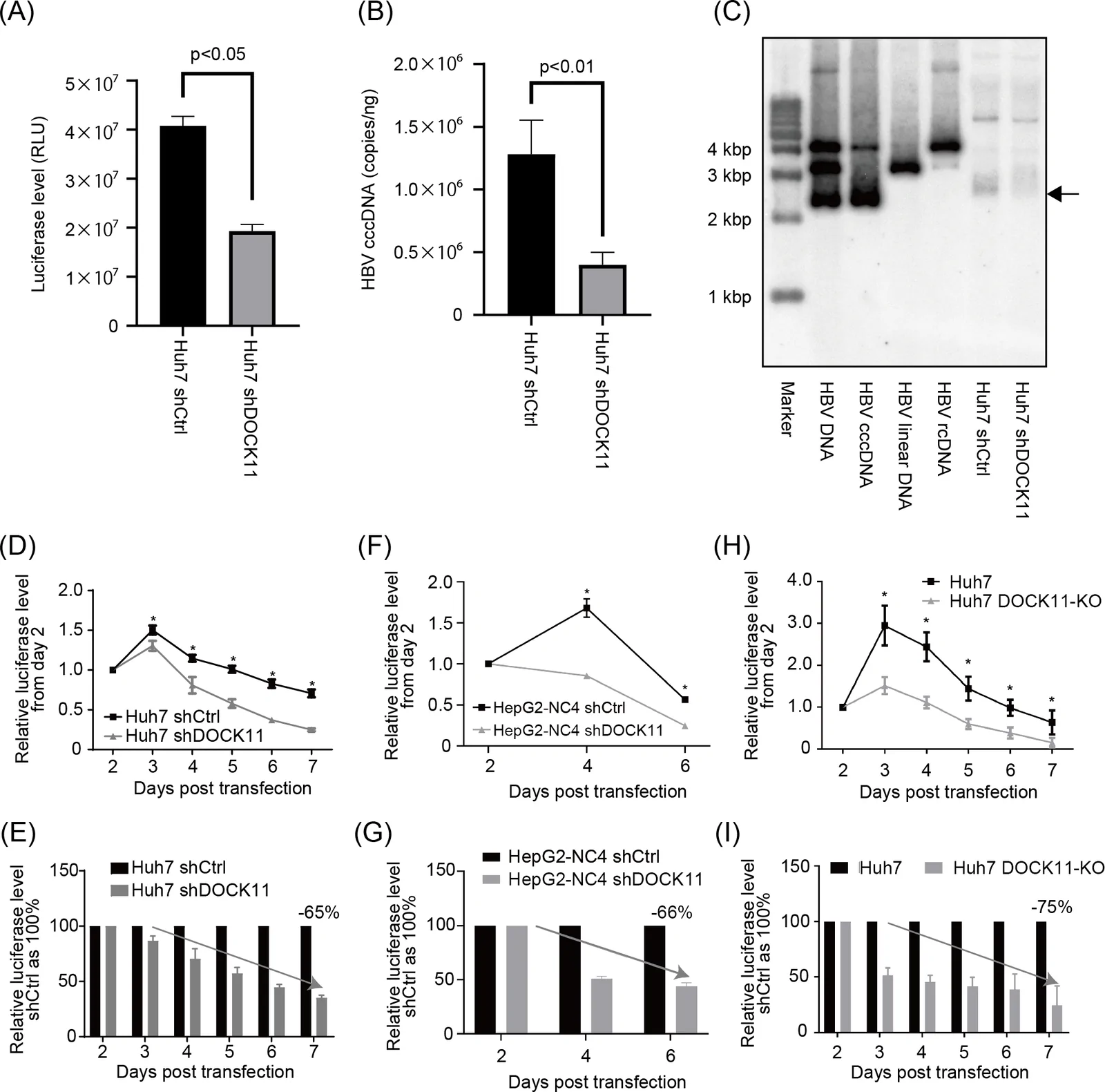
DOCK11 contributes to the maintenance of HBV cccDNA. Huh7 shCtrl and shDOCK11 cells were transfected with minicircle HBV cccDNA with a Gaussia luciferase reporter. On day 1 post-transfection, cells were reseeded at a density of 2 × 10^4^ cells per well of 96-well plates. (**A**) Luciferase activity in the culture supernatants was measured on day 6 post-transfection. A significant reduction in luciferase levels was observed in Huh7-shDOCK11 cells (*P* < 0.05). (**B**) Total DNA was extracted from the same cell lysates, and quantitative PCR for HBV cccDNA indicated a significant decrease in cccDNA levels in Huh7-shDOCK11 cells (*P* < 0.01). (**C**) Southern blotting further confirmed the reduction in HBV cccDNA in Huh7-shDOCK11 cells. The band corresponding to the expected migration of cccDNA is indicated by an arrow. HBV DNA markers shown here are plasmid-based HBV DNA surrogates generated in *Escherichia coli* and used as reference markers. (**D and E**) Time course analysis of luciferase activity from day 2 to day 7 post-transfection showed a progressive decrease in luciferase levels in Huh7-shDOCK11 cells. (**F–I**) A comparable reduction in luciferase activity was observed in HepG2-NC4 shDOCK11 cells and Huh7 DOCK11-KO cells (Fig. S1). No significant reductions in transfection efficiency, cell viability, or CMV promoter-driven luciferase activity were observed in DOCK11-depleted cells. cccDNA, covalently closed circular DNA; DOCK11, dedicator of cytokinesis 11; HepG2-NC4, HepG2-NTCP-C4; KO, knockout; PCR, polymerase chain reaction; rcDNA, relaxed circular DNA; RLU, relative light units; shCtrl, control short hairpin RNA; and shDOCK11, short hairpin RNA targeting DOCK11.

We first investigated the role of DOCK11 in HBV cccDNA maintenance using minicircle HBV cccDNA with a Gaussia luciferase reporter (mcHBV-GLuc cccDNA) (16). Luciferase activity in the culture supernatant of Huh7 cells transfected with mcHBV-Gluc cccDNA was measured at 6 days post-transfection. DOCK11 knockdown by shRNA significantly reduced luciferase activity compared to control cells (Fig. 1A). Consistent with this, quantitative PCR analysis revealed a significant decrease in HBV cccDNA levels in Huh7 DOCK11-knockdown cells (Fig. 1B). Southern blot analysis further confirmed that DOCK11 knockdown reduced HBV cccDNA levels (Fig. 1C). To evaluate sequential changes in luciferase activity, we measured the activity from days 2 to 7 post-transfection. Compared to day 2, luciferase activity gradually declined in Huh7 DOCK11-knockdown cells (Fig. 1D). When the luciferase activity in control cells was normalized to 100%, Huh7 DOCK11-knockdown cells exhibited a progressive reduction, with a 65% decrease by day 7 (Fig. 1E). Similar results were also obtained in HepG2-NTCP-C4 DOCK11-knockdown cells and Huh7 DOCK11-knockout cells (Fig. 1F through I). Notably, early luciferase activity at 2 days post-transfection was robust in both control and DOCK11-depleted cells, suggesting that DOCK11 depletion did not markedly impair transfection efficiency (Fig. S1B, F, and J). Raw luciferase data are shown in Fig. S1C, G, and K. In addition, cell viability assays showed no significant decline in DOCK11-depleted cells at later time points (Fig. S1D, H, and L). Furthermore, analysis of a cytomegalovirus (CMV) promoter-driven non-HBV reporter showed comparable *Renilla* luciferase activity kinetics between control and DOCK11-depleted cells, indicating that DOCK11 depletion does not affect CMV promoter-driven reporter expression (Fig. S1E, I, and M). These findings suggest that DOCK11 contributes to the maintenance of HBV cccDNA and also indicate a potential role in the stability of cccDNA.

### DOCK11 facilitates HBV cccDNA formation *in vitro*

**Fig 2:**
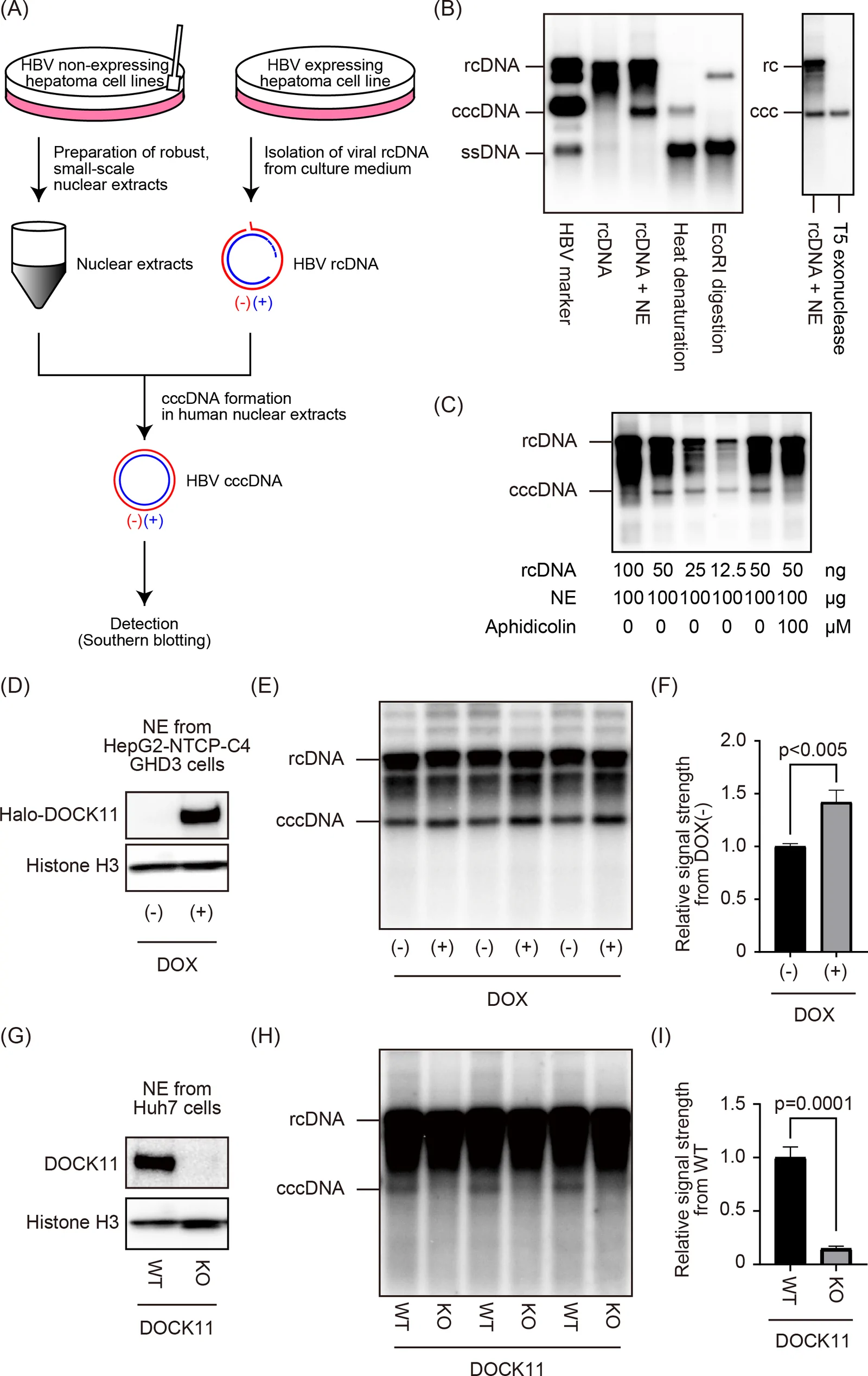
DOCK11 facilitates HBV cccDNA formation *in vitro*. (**A**) Schematic representation of the HBV cccDNA formation process in nuclear extracts. Nuclear extracts (100 μg) prepared from HBV-nonexpressing hepatoma cell lines were incubated with HBV rcDNA isolated from HBV-expressing hepatoma cells for 30 min at 37°C. The formation of cccDNA was analyzed by Southern blotting under ATP-supported reaction conditions. (**B**) HBV rcDNA was isolated from the supernatant of HepAD38 cells. Validation of *in vitro*-formed cccDNA by heat denaturation, EcoRI digestion, and T5 exonuclease treatment. (**C**) Incubation of 50 ng HBV rcDNA with 100 μg nuclear extracts resulted in robust cccDNA formation. (**D**) Nuclear extracts were prepared from HepG2-NTCP-C4 cells with and without Halo-tagged DOCK11 overexpression. (**E and F**) These nuclear extracts were incubated with HBV rcDNA, and Southern blotting demonstrated a 42% increase in cccDNA formation with DOCK11 overexpression (*P* < 0.005). (**G**) Nuclear extracts from Huh7 DOCK11-knockout cells were collected. (**H and I**) These nuclear extracts were also incubated with HBV rcDNA. Southern blotting showed that cccDNA formation was significantly suppressed by 85% in the DOCK11-knockout samples (*P* = 0.0001). ATP, adenosine triphosphate; cccDNA, covalently closed circular DNA; DOCK11, dedicator of cytokinesis 11; DOX, doxycycline; EcoRI, *Escherichia coli* restriction endonuclease RI; KO, knockout; NE, nuclear extracts; rcDNA, relaxed circular DNA; ssDNA, single-strand DNA; and WT, wild type.

To investigate the impact of DOCK11 on HBV cccDNA formation, we established an *in vitro* assay system to evaluate HBV cccDNA formation. A schematic overview of this system is presented in Fig. 2A. HBV rcDNA, isolated from the culture supernatant of HepAD38 cells (an HBV-expressing hepatoma cell line), was incubated with nuclear extracts from HBV-non-expressing hepatoma cell lines at 37°C for 30 min, leading to the formation of HBV cccDNA. The resulting HBV cccDNA was detected by Southern blotting. To validate the system, we first examined whether HBV cccDNA formed in this assay exhibited the biochemical properties of authentic cccDNA. The cccDNA generated in this system was resistant to heat denaturation (85°C for 5 min) and was converted into a linear DNA fragment upon EcoRI digestion, and it was also resistant to T5 exonuclease treatment, further confirming its circular DNA structure (Fig. 2B). Subsequently, we examined the optimal ratio of HBV rcDNA to nuclear extracts, determining that a ratio of 50 ng of HBV rcDNA to 100 µg of nuclear extract was optimal. In addition, HBV cccDNA formation at this ratio was inhibited by aphidicolin, a DNA synthesis inhibitor (Fig. 2C).

We next evaluated the role of DOCK11 in HBV cccDNA formation using this system. Nuclear extracts were collected from HepG2-NTCP-C4 cells, which allow doxycycline-inducible overexpression of Halo-tagged DOCK11 via a Tet-On system. Robust expression of Halo-DOCK11 in the nuclear extracts under overexpressing conditions was confirmed (Fig. 2D). When these nuclear extracts were used in the *in vitro* assay system, we found that overexpression of Halo-DOCK11 significantly increased HBV cccDNA formation compared to the non-overexpressing control (42% increase, *P* < 0.005) (Fig. 2E and F). Next, to further investigate the role of DOCK11 in HBV cccDNA formation, nuclear extracts were obtained from wild-type and DOCK11-knockout Huh7 cells. We performed western blotting to confirm the loss of DOCK11 expression in the knockout cells (Fig. 2G). By using these nuclear extracts in the *in vitro* assay system, we found that DOCK11 knockout markedly reduced HBV cccDNA formation (85% reduction, *P* = 0.0001) (Fig. 2H and I). Collectively, these findings demonstrate that DOCK11 facilitates HBV cccDNA formation *in vitro*.

### DOCK11 forms a complex with PARP1 during HBV cccDNA formation

**Fig 3:**
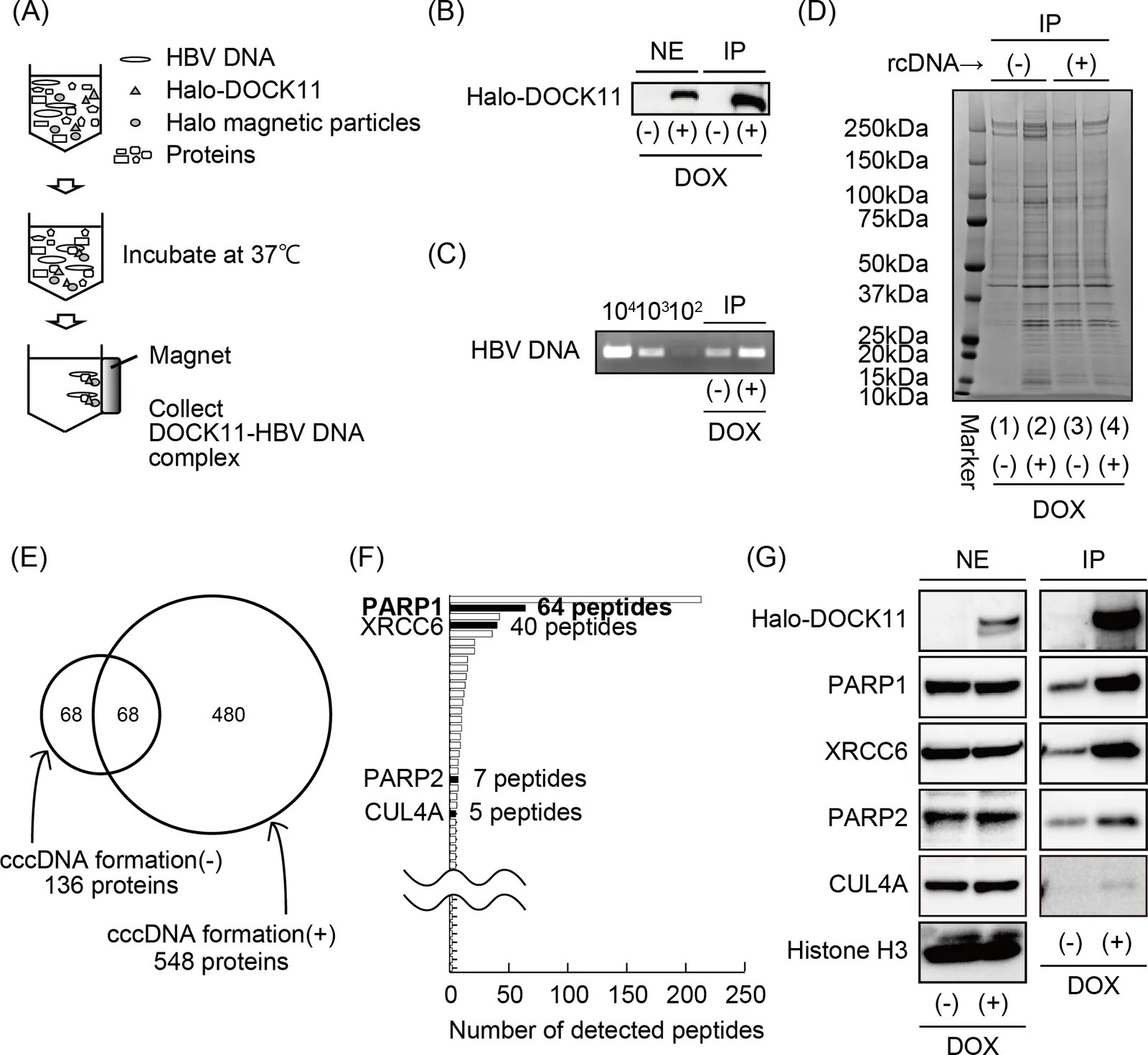
DOCK11 forms a complex with PARP1 during HBV cccDNA formation. (**A**) Immunoprecipitation of Halo-DOCK11 was performed after incubating nuclear extracts from Halo-DOCK11-overexpressing cells with HBV rcDNA. (**B**) Western blotting confirmed the successful immunoprecipitation of Halo-DOCK11. (**C**) Standard PCR of DNA extracted from the immunoprecipitates detected HBV DNA under DOCK11-overexpressing conditions. (**D**) SDS-PAGE followed by Coomassie Brilliant Blue staining revealed multiple protein bands in the immunoprecipitates. (**E**) LC-MS/MS analysis of these samples identified 68 proteins forming a complex with DOCK11, both before and after HBV cccDNA formation. (**F**) Among these proteins, four had previously been implicated in HBV cccDNA formation. (**G**) Immunoprecipitation of DOCK11 from nuclear extracts confirmed that these four candidate proteins exhibited stronger signals under DOCK11 overexpression. cccDNA, covalently closed circular DNA; CUL4A, cullin-4A; DOCK11, dedicator of cytokinesis 11; DOX, doxycycline; IP, immunoprecipitation; LC-MS/MS, liquid chromatography-tandem mass spectrometry; NE, nuclear extracts; PARP, poly(ADP-ribose) polymerase; PCR, polymerase chain reaction; rcDNA, relaxed circular DNA; SDS-PAGE, sodium dodecyl sulfate-polyacrylamide gel electrophoresis; and XRCC6, X-ray repair cross complementing 6.

To investigate the interaction between DOCK11 and rcDNA during HBV cccDNA formation, we performed immunoprecipitation with Halo magnetic particles using mixed samples for the *in vitro* HBV cccDNA formation assay. The experimental scheme is shown in Fig. 3A. Western blotting confirmed the successful overexpression of Halo-DOCK11 in the immunoprecipitated samples mixed with nuclear extracts from DOCK11-overexpressing cells (Fig. 3B). Notably, HBV DNA was strongly detected in the samples from Halo-DOCK11-overexpressing cells (Fig. 3C), suggesting that DOCK11 forms a complex with HBV DNA.

Next, to identify proteins that form complexes with DOCK11 or DOCK11-HBV DNA in the nucleus, we prepared four nuclear extracts samples: DOCK11-overexpressing and non-overexpressing extracts, each in the presence or absence of an rcDNA mixture. Following immunoprecipitation with Halo magnetic particles, each sample was analyzed by SDS-PAGE and visualized with Coomassie Brilliant Blue staining, which revealed multiple bands of varying intensities (Fig. 3D). To identify proteins co-precipitated with Halo-DOCK11, we performed liquid chromatography-tandem mass spectrometry (LC-MS/MS) analysis. For downstream analysis, we selected only proteins with high identification confidence, detection of two or more peptides, and known nuclear localization. Proteins with an abundance ratio ≥2 under DOCK11-overexpressing conditions were identified in two comparisons: in the absence of HBV cccDNA formation and in its presence. This analysis yielded 136 proteins under the absence condition and 548 proteins under the presence condition. To enrich for proteins more directly and stably associated with DOCK11, proteins detected only in the presence of HBV cccDNA were excluded, as they may reflect indirect co-precipitation mediated by HBV DNA or DNA-binding proteins. A total of 68 proteins met the abundance ratio criterion in both conditions (Fig. 3E). Of these 68 shared proteins, four—PARP1 (10), XRCC6 (17), PARP2 (18), and cullin-4A (19)—have previously been implicated in HBV cccDNA formation. These proteins were ranked according to the number of peptides detected in the LC-MS/MS analysis (Fig. 3F).

To validate these results, nuclear extracts were immunoprecipitated with Halo-DOCK11 and subjected to western blotting for the four candidate proteins. Of the four, PARP1 and XRCC6 showed results suggesting a strong interaction with DOCK11 (Fig. 3G). Based on these results, we selected PARP1, which had the highest number of detected peptides and plays a critical role in HBV cccDNA formation (10), for further investigation as the strongest potential contributor to the DOCK11-mediated promotion of HBV cccDNA formation.

### PARP1 regulates HBV cccDNA formation in an *in vitro* system

**Fig 4:**
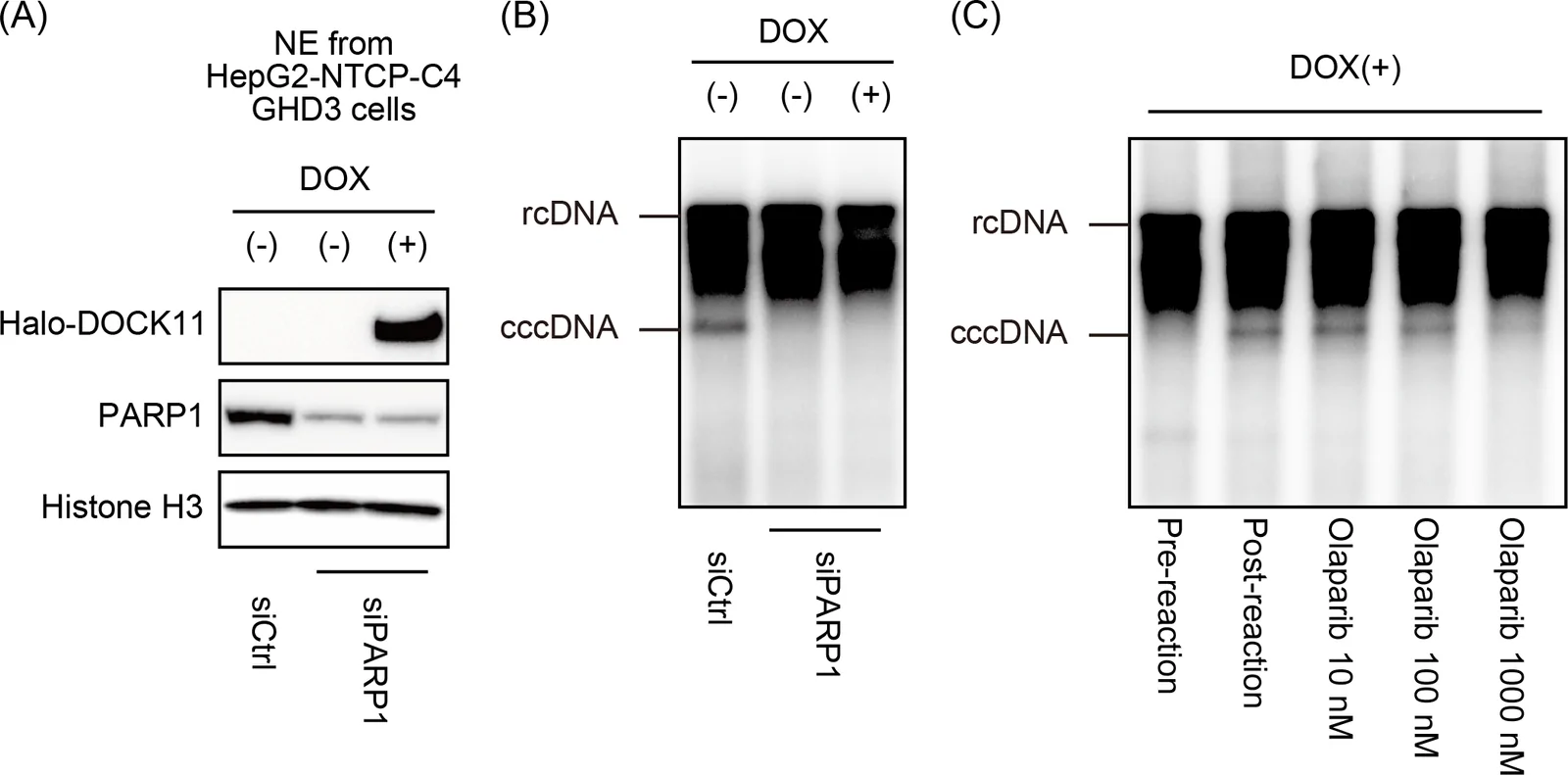
PARP1 regulates HBV cccDNA formation in an *in vitro* system. (**A**) PARP1 knockdown was performed in HepG2-NTCP-C4 Halo-DOCK11 cells using siPARP1, and the effectiveness of siPARP1 was confirmed using western blotting. (**B**) Nuclear extracts were collected from each cell type, and *in vitro* HBV cccDNA formation was performed. (**C**) Olaparib inhibited cccDNA formation under DOCK11-overexpressing conditions. “Pre-reaction” indicates a DNA sample collected immediately after rcDNA was mixed with nuclear extracts, before incubation. In contrast, “Post-reaction” indicates a DNA sample collected after incubation of rcDNA with nuclear extracts, allowing cccDNA formation. cccDNA, covalently closed circular DNA; DOCK11, dedicator of cytokinesis 11; DOX, doxycycline; NE, nuclear extracts; PARP, poly(ADP-ribose) polymerase; rcDNA, relaxed circular DNA; siCtrl, control small interfering RNA; and siPARP1, small interfering RNA targeting PARP1.

We first investigated the impact of PARP1 on HBV cccDNA formation using an *in vitro* assay system. Western blotting confirmed that PARP1 expression in nuclear extracts was reduced by PARP1 knockdown, regardless of DOCK11 expression (Fig. 4A). Under these conditions, the *in vitro* assay revealed that HBV cccDNA formation was markedly suppressed by PARP1 knockdown, even under DOCK11 overexpression (Fig. 4B).

We further evaluated the effect of PARP1 on HBV cccDNA formation using olaparib, a PARP1/2 inhibitor. Using nuclear extracts from DOCK11-overexpressing cells, an *in vitro* HBV cccDNA formation assay was performed after treating the cells with olaparib. The results demonstrated that olaparib suppressed cccDNA formation even under DOCK11-overexpressing conditions (Fig. 4C). Collectively, these findings suggest that PARP1 is required for efficient HBV cccDNA formation in this *in vitro* system.

### DOCK11 and PARP1 are closely associated in the nucleus

**Fig 5:**
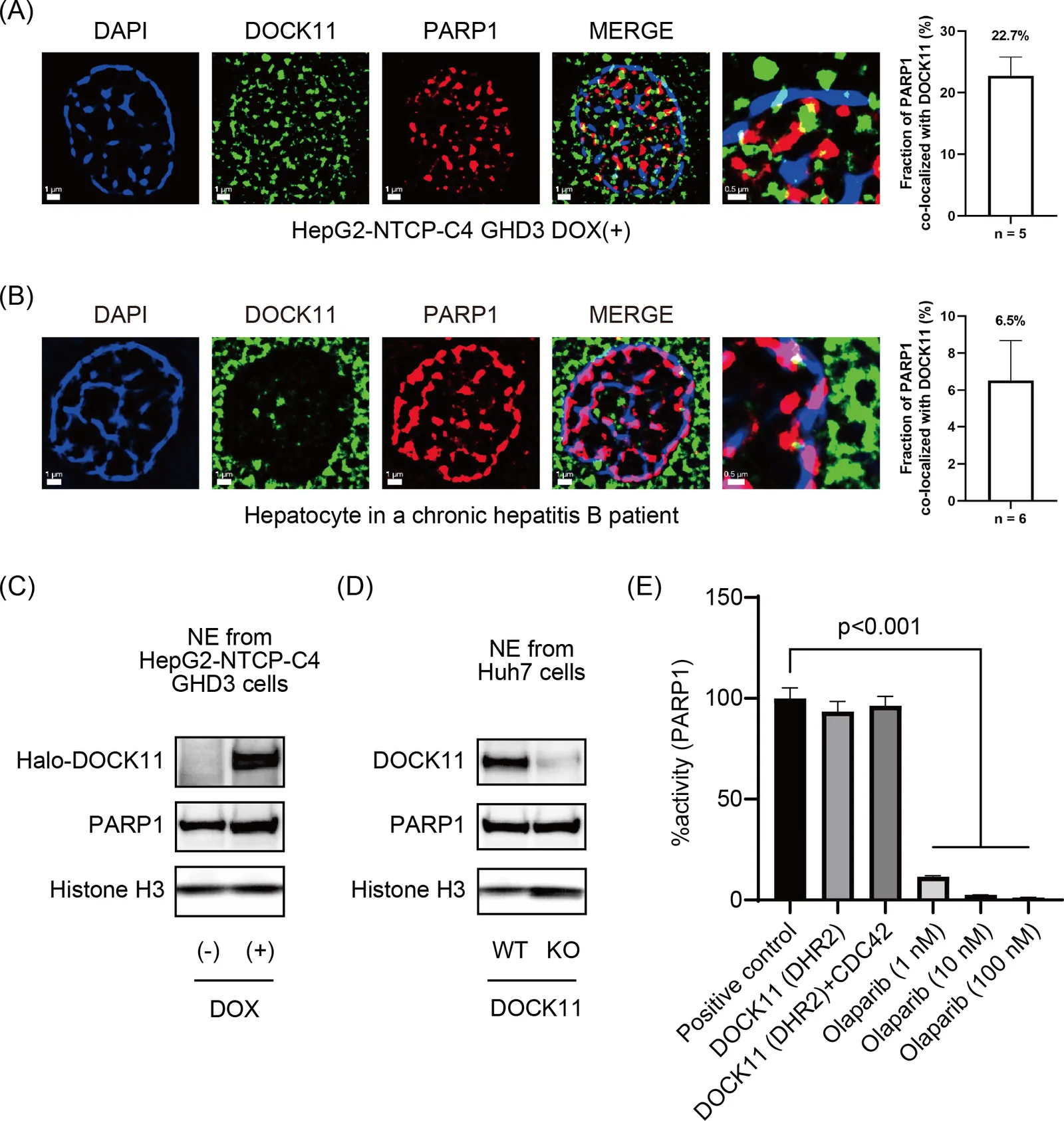
DOCK11 and PARP1 are closely associated in the nucleus. (**A**) Confocal microscopy images of HepG2-NTCP-C4 Halo-DOCK11-overexpressing cells stained with DAPI (blue), DOCK11 (green), and PARP1 (red) revealed colocalization of DOCK11 and PARP1 in the nucleus, with 22.7% of PARP1 colocalized with DOCK11. (**B**) Colocalization of DOCK11 (green) and PARP1 (red) was observed in the nuclei of hepatocytes from a patient with chronic hepatitis B, with 6.5% of PARP1 co-localized with DOCK11. (**C**) Overexpression of DOCK11 in HepG2-NTCP-C4 Halo-DOCK11 cells had no effect on endogenous PARP1 expression. (**D**) Similarly, no change in endogenous PARP1 expression was detected in Huh7 DOCK11-knockout cells. (**E**) Recombinant DOCK11 (DHR2 domain) and activated CDC42 did not alter PARP1 activity. CDC42, cell division cycle 42; DAPI, 4′,6-diamidino-2-phenylindole; DHR2, DOCK homology region 2; DOCK11, dedicator of cytokinesis 11; DOX, doxycycline; KO, knockout; NE, nuclear extracts; PARP, poly(ADP-ribose) polymerase; and WT, wild type.

To confirm the intranuclear localization of DOCK11 and PARP1, we performed super-resolution microscopy using HepG2-NTCP-C4 cells overexpressing Halo-DOCK11. Multiplex immunofluorescence staining for DOCK11 and PARP1 revealed their nuclear colocalization. A total of two images and five cells were analyzed, and all cells showed similar expression patterns. Quantitative analysis indicated that 22.7% of PARP1 was co-localized with DOCK11 (Fig. 5A). We next prepared liver tissue from a patient with chronic hepatitis B. Similar to the findings in cultured cells, DOCK11 and PARP1 were colocalized within the nucleus. A total of two images and six cells were analyzed, and all cells showed similar expression patterns. In these samples, 6.5% of PARP1 was co-localized with DOCK11 (Fig. 5B). These results suggest that DOCK11 and PARP1 are closely associated in the nucleus of both cultured cells and human liver tissue infected with HBV.

We further investigated the relationship between PARP1 and DOCK11 and found that DOCK11 overexpression did not alter endogenous PARP1 expression (Fig. 5C). Similarly, DOCK11 knockout had no effect on endogenous PARP1 expression (Fig. 5D). These findings suggest that DOCK11 does not regulate PARP1 expression. We then examined the effect of DOCK11 on PARP1 activity. Recombinant DOCK11 (DOCK homology region 2 [DHR2] domain) and recombinant CDC42 were added to a PARP1 chemiluminescent assay kit to measure PARP1 activity. The results showed that, while olaparib significantly inhibited PARP1 activity, neither DOCK11 (DHR2 domain) nor activated CDC42 altered PARP1 activity (Fig. 5E).

### DOCK11 possibly regulates the recruitment of PARP1 to DNA damage sites and HBV rcDNA

**Fig 6:**
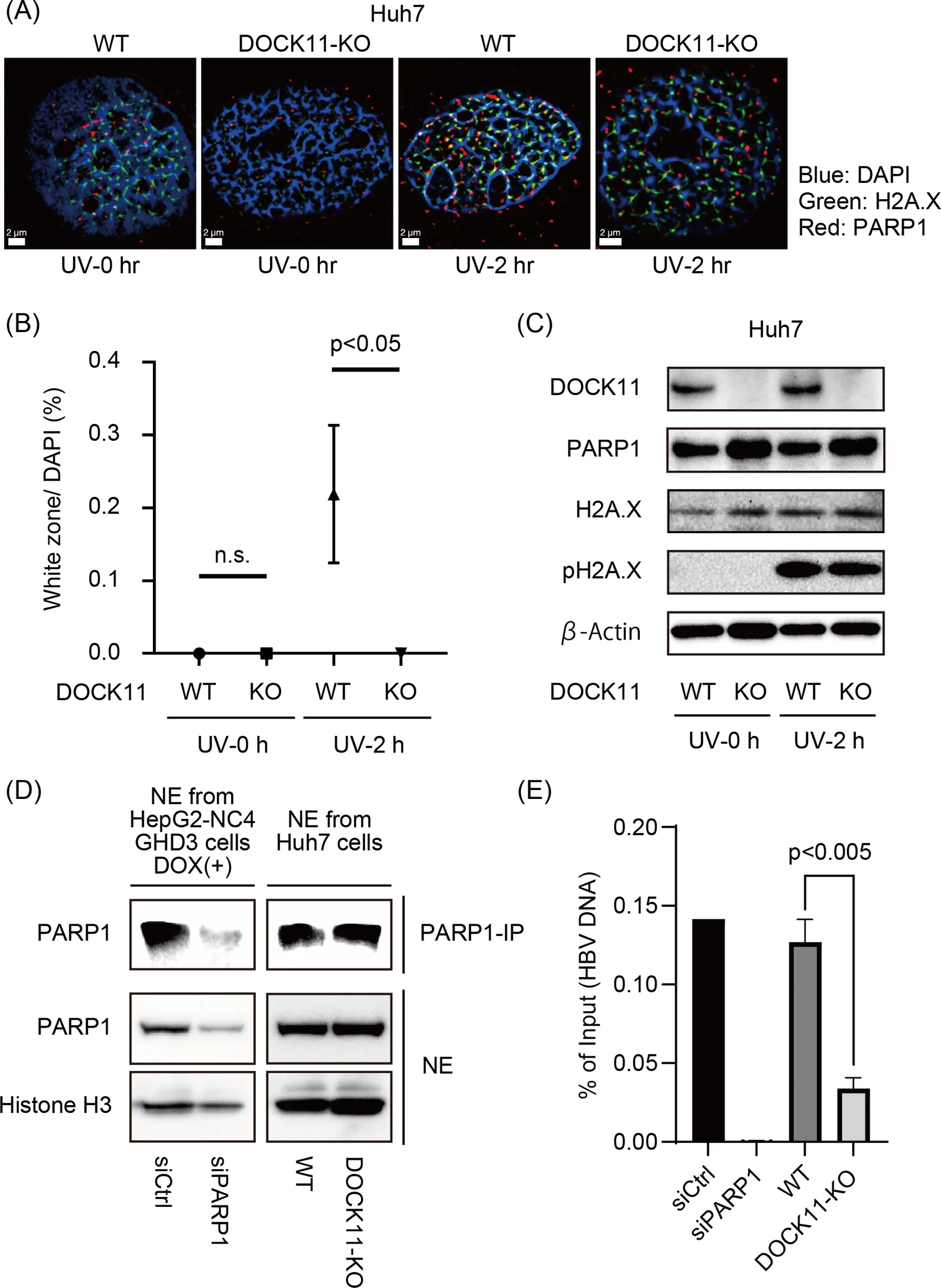
DOCK11 possibly regulates the recruitment of PARP1 to DNA damage sites and HBV rcDNA. (**A and B**) Huh7 and Huh7 DOCK11-knockout cells were subjected to UV irradiation. Cells were stained with DAPI (blue), H2A.X (green), and PARP1 (red) and analyzed using confocal microscopy. In Huh7 cells, UV irradiation promoted the colocalization of H2A.X and PARP1 in DAPI-stained nuclei at 2 h post-irradiation, while this colocalization was significantly reduced in Huh7 DOCK11-KO cells (*P* < 0.05, *n* = 3). (**C**) Western blotting was performed using cell lysates from WT and DOCK11-KO cells with or without UV irradiation to examine the expression levels of DOCK11, PARP1, H2A.X, phosphorylated H2A.X, and ACTB. Total PARP1 protein levels were comparable between WT and DOCK11-KO cells. Phosphorylated H2A.X induction was confirmed at 2 h post-irradiation. (**D**) Following HBV cccDNA formation, nuclear extracts were prepared and subjected to immunoprecipitation using a PARP1 antibody and magnetic beads. As positive and negative controls, nuclear extracts from siCtrl- and siPARP1-transfected HepG2-NTCP-C4 Halo-DOCK11 cells were included. Nuclear extracts from Huh7 and Huh7 DOCK11-KO cells were analyzed in parallel. Comparable immunoprecipitation efficiency of PARP1 was confirmed in both WT and DOCK11-KO conditions. (**E**) DNA was extracted from the PARP1 immunoprecipitates using phenol-chloroform extraction followed by ethanol precipitation, and HBV DNA was quantified by quantitative PCR. DOCK11 knockdown significantly reduced the association between PARP1 and HBV DNA (*P* < 0.005). ACTB, β-actin; cccDNA, covalently closed circular DNA; DAPI, 4′,6-diamidino-2-phenylindole; DOCK11, dedicator of cytokinesis 11; H2A.X, H2A.X variant histone; HepG2-NC4, HepG2-NTCP-C4; IP, immunoprecipitation; KO, knockout; NE, nuclear extracts; PARP1, poly(ADP-ribose) polymerase 1; PCR, polymerase chain reaction; pH2A.X, phosphorylated H2A.X; siCtrl, control small interfering RNA; siPARP1, PARP1-targeting small interfering RNA; UV, ultraviolet; and WT, wild type.

PARP1 is a key player in the DNA damage response, initiating DNA repair upon binding to damaged DNA. We therefore investigated the effect of DOCK11 on the recruitment of PARP1 to DNA damage sites. DNA damage was induced by UV irradiation in both wild-type Huh7 cells and DOCK11-knockout Huh7 cells. Multiplex immunofluorescence staining for H2A.X and PARP1 was performed at 0 and 2 h after UV exposure. In wild-type Huh7 cells, UV irradiation increased the colocalization of H2A.X and PARP1 in 4′,6-diamidino-2-phenylindole (DAPI)-stained nuclei from 0% to 0.22%. In contrast, the UV-induced increase in the colocalization of H2A.X and PARP1 was barely observed in DOCK11-knockout cells (*P* < 0.05) (Fig. 6A and B). Western blotting confirmed that DOCK11 knockout did not affect PARP1 expression or the UV-induced expression of phosphorylated H2A.X (pH2A.X) (Fig. 6C). We next evaluated whether DOCK11 affects the binding of PARP1 to rcDNA. Nuclear extracts collected from wild-type and DOCK11-knockout Huh7 cells were incubated with rcDNA, followed by immunoprecipitation using an anti-PARP1 antibody. Successful precipitation of PARP1 was confirmed by Western blotting (Fig. 6D). DNA was extracted from the immunoprecipitated samples, and quantitative PCR for HBV DNA revealed a significant reduction in HBV DNA levels in DOCK11-knockout cells (*P* < 0.005) (Fig. 6E). These results suggest that DOCK11 possibly regulates the recruitment of PARP1 to DNA damage sites and HBV rcDNA.

## DISCUSSION

The formation and maintenance of HBV cccDNA within the nuclei of hepatocytes play a central role in the persistence of HBV infection (20). HBV cccDNA is formed from HBV rcDNA in the nucleus and maintained as a minichromosome that serves as the primary template for HBV transcription (21, 22). The complete elimination of HBV cccDNA remains challenging with current therapies. Therefore, a deeper understanding of the mechanisms underlying cccDNA formation and maintenance, particularly the contribution of host factors, is crucial for the development of curative therapies (23–25). We previously identified DOCK11 as a critical host factor in the maintenance of HBV. In this study, we aimed to elucidate the role of DOCK11 in HBV cccDNA formation.

We first utilized the mcHBV-GLuc reporter system (16), a surrogate for HBV cccDNA, to assess the role of DOCK11 in its maintenance. As expected, the results demonstrated that DOCK11 is indeed involved in maintaining HBV cccDNA. Importantly, as described in the previous report (16), the mcHBV-GLuc system reflects the expression of input cccDNA rather than amplification of the cccDNA pool via intracellular recycling pathways. Thus, the reduced signals observed in DOCK11-depleted cells may also reflect a potential role for DOCK11 in cccDNA stability. In addition, an *in vitro* HBV cccDNA formation assay revealed that DOCK11 contributes to HBV cccDNA formation. It should be noted that five core components, including LIG1, FEN1, POLδ, RFC, and PCNA, have been reported to be sufficient to support HBV cccDNA formation *in vitro* (2, 3). For the cccDNA formation assays shown in Fig. 2, experimental systems were selected based on endogenous DOCK11 expression levels, with overexpression performed in HepG2-derived cells and loss-of-function analyses conducted in Huh7 cells, where DOCK11 expression is relatively higher. Our assay system was optimized to detect relatively small amounts of newly formed cccDNA; therefore, a reduction in cccDNA formation efficiency may result in signals falling below the detection limit. Thus, the markedly reduced cccDNA signals observed under DOCK11-deficient and PARP1-knockdown conditions do not necessarily indicate a complete abrogation of cccDNA formation, but rather a substantial decrease in formation efficiency. Importantly, our *in vitro* assay system specifically focuses on the conversion step from rcDNA to cccDNA and does not recapitulate the entire HBV life cycle. Nevertheless, the consistent findings obtained across our previous infection models and the present cccDNA formation assays underscore the physiological relevance of DOCK11.

We next used immunoprecipitation with Halo magnetic particles to investigate the mechanism by which DOCK11 regulates HBV cccDNA formation, finding that DOCK11 forms a complex with HBV DNA. Based on our previous finding that DOCK11 does not appear to directly associate with HBV cccDNA (15), we hypothesized that the complex contains an intermediary protein that directly associates with HBV cccDNA. To identify potential protein candidates, we conducted LC-MS/MS analysis, a useful method for identifying proteins in multiprotein complexes (26). Using the immunoprecipitated samples, we identified 68 proteins that may have formed complexes with DOCK11, regardless of the presence of HBV DNA. These proteins included not only those related to actin dynamics, a known function of DOCK11, but also several DNA repair-related proteins, including CDC42-related factors such as Lamina-associated polypeptide 2, ARF GTPase-activating protein GIT2, and Actin nucleation-promoting factor WASL, which support the reliability of our LC-MS/MS analysis. Among the proteins previously implicated in HBV cccDNA formation, such as PARP1 (10), XRCC6 (17), PARP2 (18), and cullin-4A (19), PARP1 was detected with the highest number of peptides in LC-MS/MS. Although HBV DNA was detected in complexes containing DOCK11, our previous work and the present data do not support a model in which DOCK11 directly binds HBV cccDNA. Rather, these findings suggest that DOCK11 may be part of a multiprotein complex that associates with HBV DNA through intermediary factors. PARP1 acts as a sensor in multiple DNA damage response pathways, including single-strand break repair, nucleotide excision repair, homologous recombination, non-homologous end joining (NHEJ), and mismatch repair (27, 28). When PARP1 senses DNA strand breaks, it undergoes auto-poly-ADP-ribosylation using NAD^+^ as a substrate, which facilitates the recruitment of DNA repair factors and induces chromatin remodeling (29). Several studies have reported associations between PARP1 and HBV. For example, during the early stages of infection, HBV induces oxidative DNA damage in the host hepatocyte genome and exploits the PARP1-mediated NHEJ pathway for viral DNA integration (30). In addition, a PARP1-binding motif has been identified within the HBV core promoter, contributing to enhanced viral replication efficiency (31). PARP1 has also been shown to bind HBV rcDNA, promoting the activation of other DNA repair proteins and facilitating the formation of cccDNA (10). Consistent with previous reports, our *in vitro* HBV cccDNA formation assay demonstrated that knockdown of PARP1 and olaparib markedly suppressed cccDNA formation.

Because no previous studies have reported a link between DOCK11 and PARP1, we explored their potential relationship. We first observed that DOCK11 and PARP1 are colocalized in the nuclei of both cultured cells and human liver tissue. We also confirmed that altering the expression of DOCK11 did not affect the endogenous PARP1 expression level. We then showed that recombinant DOCK11 and activated CDC42 did not affect PARP1 activity. Importantly, the ability of DOCK11 to promote HBV cccDNA formation was attenuated by PARP1 knockdown and PARP1/2 inhibition, indicating that DOCK11 acts in a PARP1-dependent manner rather than functioning as an alternative or upstream determinant of cccDNA formation. Furthermore, while PARP1 is predominantly localized in the nucleus, DOCK11 is distributed in both the cytoplasm and the nucleus in Huh7 cells and HepG2-NTCP-C4 Halo-DOCK11 cells (Fig. S2). DOCK11 knockout decreased the UV-induced colocalization of H2A.X and PARP1 in DAPI-stained chromatin and the association of PARP1 with HBV rcDNA. These findings suggest that DOCK11 may facilitate the recruitment of PARP1 to DNA damage sites. Although the precise mechanism underlying this recruitment remains unclear, recent studies have demonstrated that nuclear actin plays a crucial role in the recruitment and regulation of DNA repair factors. For example, destabilization of actin filaments impairs the accumulation of key DNA damage response proteins such as XRCC1 and PCNA at damage sites (32). Moreover, DOCK11 has been reported to promote nuclear actin polymerization and activate the ATR–Chk1 signaling pathway (14). Collectively, these observations support a model in which DOCK11 may indirectly facilitate the recruitment or retention of PARP1 at DNA damage sites, potentially through modulation of nuclear actin dynamics.

**Fig 7:**
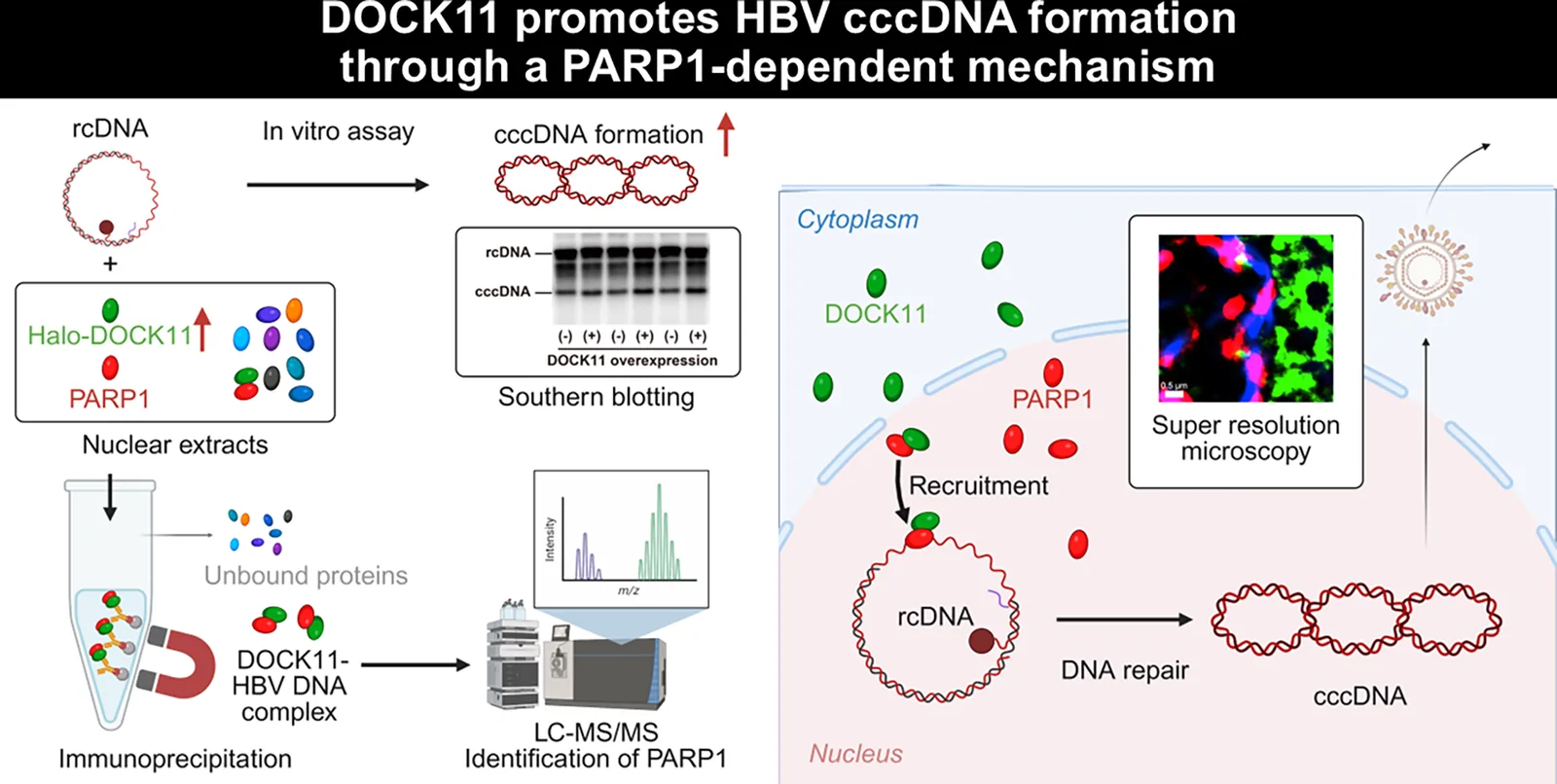
Schematic summary.

In this study, we demonstrated that DOCK11 forms a complex with HBV DNA and PARP1, thereby supporting HBV cccDNA formation (shown in Fig. 7). These insights highlight the therapeutic potential of targeting the host factor DOCK11 in the development of novel antiviral strategies aimed at eliminating HBV cccDNA, which remains a major obstacle to achieving a cure for chronic hepatitis B.

## MATERIALS AND METHODS

Some Materials and Methods are detailed in the supplemental material, including information on cell lines (Table S1), the antibodies used for western blotting and immunofluorescence staining (Table S2), the primer sets for HBV DNA (Table S3), siRNAs (Table S4), PARP1/2 inhibitor (olaparib) (Table S4), and 68 proteins identified by liquid chromatography-mass spectrometry (Table S5).

### Minicircle HBV cccDNA with a Gaussia luciferase reporter

To evaluate the maintenance of HBV cccDNA, we used mcHBV-GLuc cccDNA (16). mcHBV-GLuc cccDNA mimics HBV cccDNA and allows for the convenient quantification of temporal cccDNA levels via luciferase activity. We transfected the mcHBV-GLuc cccDNA into cultured cells using Lipofectamine 3000 Reagent (Thermo Fisher Scientific). Then, we measured luciferase activity by adding substrate to the collected culture supernatant and analyzing the signal using a GloMax Discover Microplate Reader (Promega).

To evaluate whether DOCK11 depletion affected transfection efficiency or cell viability, early luciferase activity and cell viability were assessed. At 1 day post-transfection, equal numbers of viable cells (2 × 10^4^ cells per well) were re-seeded into 96-well plates to normalize cell numbers across conditions. Luciferase activity was measured at 2 days post-transfection, and cell viability was assessed at days 4 and 6 using the Cell Counting Kit-8 (Dojindo). Absorbance was measured at 450 nm according to the manufacturer’s instructions.

For the control assay, cells were transfected with a CMV promoter-driven *Renilla* luciferase vector (pCMV-Green *Renilla* Luc; Thermo Fisher Scientific), and luciferase activity was measured at days 2, 4, and 6. Data were normalized to day 2 values to assess the temporal stability of CMV promoter-driven reporter expression.

### Quantitative PCR

HBV cccDNA levels following mcHBV-GLuc cccDNA transfection were assessed using DNA extracted from cultured cells. The extracted DNA samples were treated with ATP-dependent DNase to selectively digest linear and relaxed circular HBV DNA while sparing cccDNA. Subsequently, the cccDNA-enriched DNA fraction was quantified by qPCR using HBV DNA-specific primers listed in Table S3.

### Southern blotting

To investigate the amount of HBV cccDNA, we performed Southern blotting using the radioactive isotope ^32^P. HBV DNA markers shown in Fig. 1C and 2B are plasmid-based HBV DNA surrogates generated in *Escherichia coli* and were used as reference markers rather than authentic HBV DNA. Signals were detected using the Typhoon FLA 7000 laser scanner (GE Healthcare). The authors conducting the experiments involving radioisotopes had completed the required training for handling such materials. These experiments were conducted at the Central Institute of Radioisotope Science, Advanced Science Research Center, Kanazawa University.

### HBV rcDNA preparation

HBV rcDNA for *in vitro* HBV cccDNA formation was prepared based on the method described by Lewellyn and Loeb (33). Culture supernatants containing HBV virions from HepAD38 cells were collected through centrifugation and polyethylene glycol precipitation. The precipitates were treated with micrococcal nuclease to remove non-encapsidated nucleic acids, followed by proteinase K digestion to remove viral capsid proteins. HBV rcDNA was then extracted with phenol-chloroform and collected by ethanol precipitation.

### Preparation of nuclear extracts

Nuclear extracts for *in vitro* HBV cccDNA formation were prepared according to the method described by Folco et al. (34) to preserve nuclear factors involved in DNA metabolism and repair processes. Cultured cells were harvested using a cell scraper and swollen in a hypotonic buffer to facilitate nuclear isolation. Nuclei were isolated by Dounce homogenization and centrifugation, followed by concentration and dialysis to remove small molecules and adjust buffer conditions. The dialysis of nuclear extracts was conducted using an Xpress Micro Dialyzer MD300 (Scienova). After preparation, nuclear extracts were aliquoted and stored at −80°C, and only the required amount was thawed for each experiment to avoid repeated freeze–thaw cycles. The quality and functionality of nuclear extracts were validated by their ability to support reproducible *in vitro* HBV cccDNA formation, and only extracts that consistently supported cccDNA formation were used for subsequent analyses, including immunoprecipitation and liquid chromatography-based analyses.

### *In vitro* HBV cccDNA formation

We conducted *in vitro* HBV cccDNA formation based on the methods described by Wei and Ploss (2, 3). Briefly, HBV rcDNA was incubated with nuclear extracts in a cell-free system designed to mimic the nuclear environment. The reaction mixture contained ATP, deoxynucleotide triphosphates, and creatine kinase to support enzymatic reactions required for rcDNA repair and conversion into cccDNA. Reactions were incubated at 37°C for 30 min to form HBV cccDNA.

### Immunoprecipitation and liquid chromatography-tandem mass spectrometry

Immunoprecipitation of Halo-DOCK11 was performed using Helo-Trap Magnetic Particles M-270 (Proteintech Group, Inc.). The immunoprecipitated samples were analyzed using LC-MS/MS, conducted at the Division of Life Science, Graduate School of Natural Science and Technology, Kanazawa University. Among the proteins detected in LC-MS/MS, we selected those meeting the following criteria as reliable nuclear proteins for analysis: a protein false-discovery rate confidence categorized as “high,” detection of at least two peptides, and known localization to the nucleus.

Immunoprecipitation of PARP1 was performed using a PARP1 antibody (F-2; sc-8007, Santa Cruz Biotechnology, Inc.) and Protein G Mag Sepharose (Cytiva). The immunoprecipitated samples were confirmed by Western blotting and further analyzed by quantitative PCR for HBV DNA following DNA extraction.

### Multiplex immunofluorescence staining

Multiplex immunofluorescence staining was performed to simultaneously visualize two different proteins. HepG2-NTCP-C4 Halo-DOCK11 cells or human liver tissue were fixed, permeabilized, and incubated with Alexa Fluor 488-conjugated polyclonal DOCK11 antibody (GTX55982, GeneTex) and CoraLite Plus 647-conjugated PARP1 polyclonal antibody (CL647-13371, Proteintech). Huh7 cells were incubated with primary antibodies, and Alexa Fluor 488 and Alexa Fluor 594 (Thermo Fisher Scientific) were used as fluorescent labels. Stained cells were observed at room temperature in a dark room using a Dragonfly 200 confocal microscope (Oxford Instruments). Image processing was performed using Imaris Viewer (Oxford Instruments), with brightness and contrast adjusted to enhance image clarity.

### PARP1 activity

To assess PARP1 activity, we used a PARP1 Chemiluminescent Assay Kit (BPS BioScience). A histone-coated 96-well plate was incubated with PARP1 enzyme, an activated DNA template, and a biotinylated NAD^+^ substrate mixture in optimized assay buffer. After incubation, the plate was treated with streptavidin-HRP and an ELISA ECL substrate to generate chemiluminescence, which was measured to assess PARP1 activity. We used olaparib, a PARP1/2 inhibitor, as a negative control. Recombinant DOCK11 and CDC42 were used as described in a previous report (13).

### Statistical analysis

Data from the luciferase assay, qPCR, and PARP1 activity assays were analyzed with an unpaired Student’s *t*-test (*n* = 3–5) using GraphPad Prism version 8 for Windows (GraphPad Software). Southern blotting signals were quantified using ImageJ software (Wayne Rasband, NIH) and statistically analyzed with an unpaired *t*-test (*n* = 3) using GraphPad Prism. The co-stained area in immunofluorescence images was quantified using ImageJ and analyzed with an unpaired *t*-test (*n* = 3) using GraphPad Prism. In all statistical analyses, *P* < 0.05 was considered significant.

## Data Availability

The LC-MS/MS data supporting the results of this study are available from Figshare at https://doi.org/10.6084/m9.figshare.31368130.

## SUPPLEMENTAL MATERIAL

Supplemental figures and tables spectrum.04140-25-s0001.docx Figure S1: Shows transfection efficiency, cell viability, and CMV promoter-driven control reporter stability in control and DOCK11-depleted cells. Figure S2: shows cytoplasmic and nuclear fractions analyzed by Western blotting in hepatoma cell lines. Table S1: Information on cell lines. Table S2: The antibodies used for western blotting and immunofluorescence staining. Table S3: The primer sets for HBV DNA. Table S4: siRNAs, poly(ADP-ribose) polymerase 1/2 (PARP1/2) inhibitor (olaparib). Table S5: 68 proteins identified by liquid chromatography-mass spectrometry.

ASM does not own the copyrights to Supplemental Material that may be linked to, or accessed through, an article. The authors have granted ASM a non-exclusive, world-wide license to publish the Supplemental Material files. Please contact the corresponding author directly for reuse.
