# Supplementary material for: DOCK11 promotes HBV cccDNA formation through a PARP1-dependent mechanism
Source: Microbiol Spectr. 2026 Jun 16;14(7):e04140-25. doi: 10.1128/spectrum.04140-25 (PMC13340184; doi:10.1128/spectrum.04140-25)
Supplement: Supplemental figures and tables — Figure S1: Shows transfection efficiency, cell viability, and CMV promoter-driven control reporter stability in control and DOCK11-depleted cells. Figure S2: shows cytoplasmic and nuclear fractions analyzed by Western blotting in hepatoma cell lines. Table S1: Information on cell lines. Table S2: The antibodies used for western blotting and immunofluorescence staining. Table S3: The primer sets for HBV DNA. Table S4: siRNAs, poly(ADP-ribose) polymerase 1/2 (PARP1/2) inhibitor (olaparib). Table S5: 68 proteins identified by liquid chromatography-mass spectrometry. [file spectrum.04140-25-s0001.docx]

**Supplemental Files**

Title of the paper: DOCK11 promotes HBV cccDNA formation through a PARP1-dependant mechanism

First Author Name: Hideo Takayama, MD.

Supplemental Figure 1 shows transfection efficiency, cell viability, and CMV promoter–driven control reporter stability in control and DOCK11-depleted cells. Supplemental Figure 2 shows cytoplasmic and nuclear fractions analyzed by Western blotting in hepatoma cell lines. Information on cell lines (Supplemental Table 1), the antibodies used for western blotting and immunofluorescence staining (Supplemental Table 2), the primer sets for HBV DNA (Supplemental Table 3), siRNAs (Supplemental Table 4), poly(ADP-ribose) polymerase 1/2 (PARP1/2) inhibitor (olaparib) (Supplemental Table 4), and 68 proteins identified by liquid chromatography-mass spectrometry (Supplemental Table 5) is provided in this Supplemental Files.

Supplemental Figure 1

**
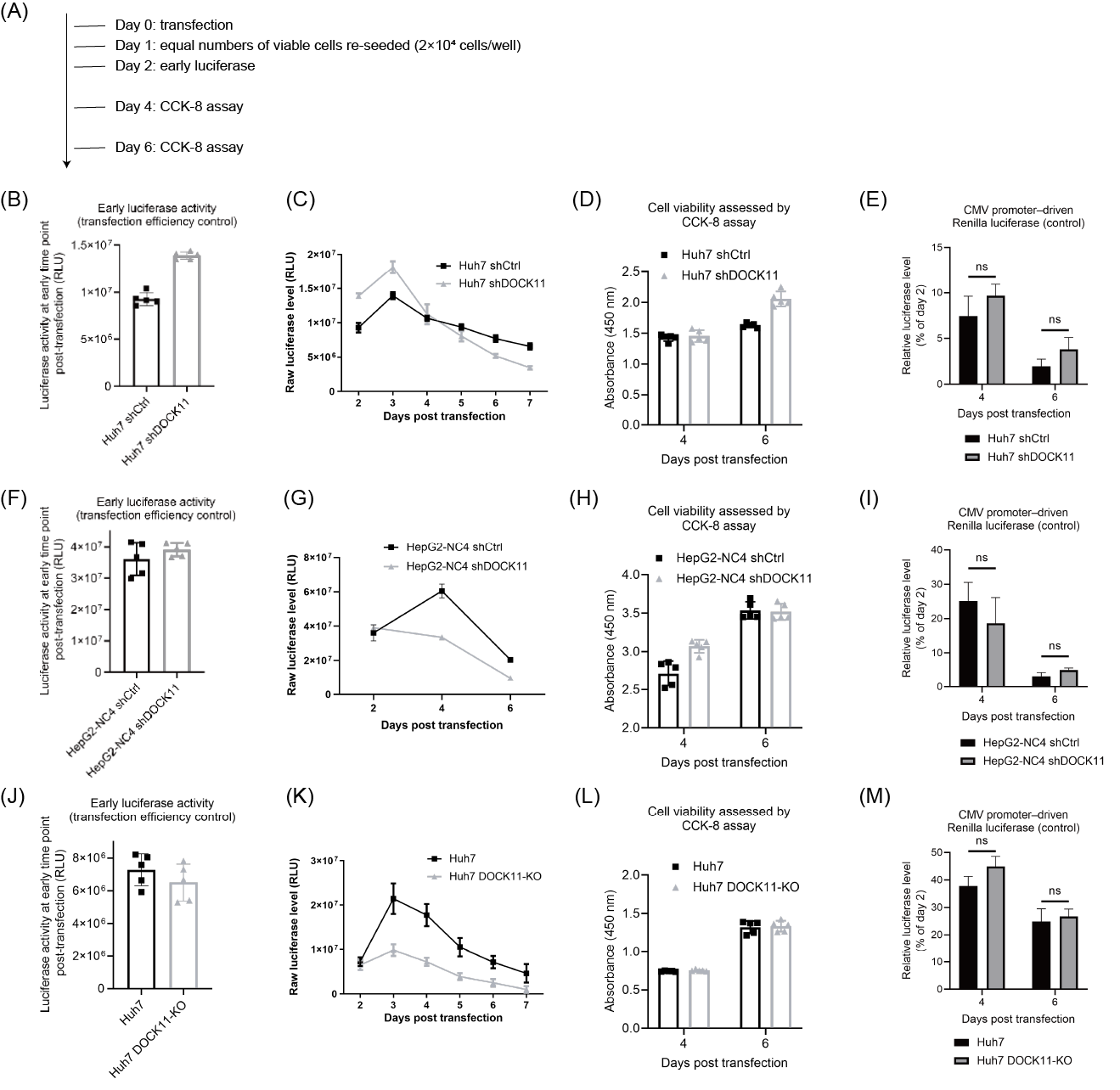
**

Supplemental Figure 1. Transfection efficiency, cell viability, and CMV promoter–driven control reporter stability in control and DOCK11-depleted cells

(A) To exclude the possibility that reduced mcHBV-derived signals in DOCK11-depleted cells resulted from differences in transfection efficiency or cell viability, early luciferase activity and cell viability assays were performed. At 1 day post-transfection, equal numbers of viable cells (2 × 10⁴ cells per well) were re-seeded. (B, F, and J) Early luciferase activity was measured at 2 days post-transfection and used as a surrogate marker for transfection efficiency, showing robust expression in both control and DOCK11-depleted cells. (C, G, and K) Time-course of raw luciferase activity (RLU) across all samples. (D, H, and L) Cell viability was evaluated using the CCK-8 assay at 4 and 6 days post-transfection, and no significant decrease was observed in DOCK11-depleted cells compared with control cells. Absorbance was measured at 450 nm. (E, I, and M) Time-course analysis of CMV promoter–driven Renilla luciferase activity normalized to day 2 values (% of day 2). No significant differences in the rate of signal decline were observed between control and DOCK11-depleted cells. CCK-8, Cell Counting Kit-8; DOCK11, dedicator of cytokinesis 11; HepG2-NC4, HepG2-NTCP-C4; KO, knockout; RLU, relative light units; shCtrl, short hairpin control; shDOCK11, short hairpin targeting DOCK11.

Supplemental Figure 2


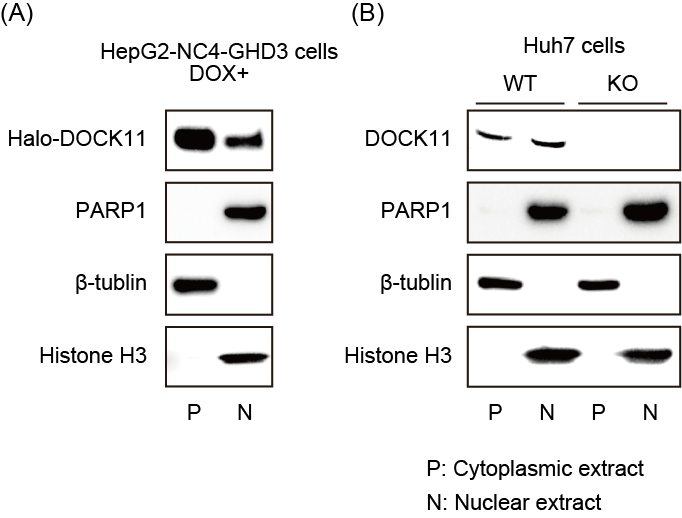


Supplemental Figure 2. Western blot analysis of cytoplasmic and nuclear fractions in hepatoma cell lines

Cytoplasmic and nuclear extracts were prepared using the NE-PER™ Nuclear and Cytoplasmic Extraction Kit (Thermo Fisher Scientific). (A) Cytoplasmic and nuclear extracts were collected from HepG2-NTCP-C4 Halo-DOCK11-overexpressing cells. Western blotting was performed using antibodies against Halo, PARP1, β-tubulin, and Histone H3. PARP1 was predominantly detected in the nuclear fraction, whereas DOCK11 was present in both cytoplasmic and nuclear fractions. (B) The same fractionation and Western blot analysis were performed in Huh7 cells and Huh7 DOCK11-KO cells, yielding similar results. DOCK11, dedicator of cytokinesis 11; DOX, doxycycline; HepG2-NC4, HepG2-NTCP-C4; KO, knockout; PARP1, poly(ADP-ribose) polymerase 1; WT, wild type.

Supplemental Table 1. Cell lines.

| Cell line name | Description |
| --- | --- |
| Huh7 shCtrl cells | Described in Li YY, et al., Cell Mol Gastroenterol Hepatol 2023 (1). |
| Huh7 shDOCK11 cells | Described in Li YY, et al., Cell Mol Gastroenterol Hepatol 2023 (1). |
| HepG2-NTCP-C4 shCtrl cells | Described in Li YY, et al., Cell Mol Gastroenterol Hepatol 2023 (1). |
| HepG2-NTCP-C4 shDOCK11 cells | Described in Li YY, et al., Cell Mol Gastroenterol Hepatol 2023 (1). |
| Huh7 cells | Authenticated by DNA fingerprinting in 2016. |
| Huh7 DOCK11-KO cells | Huh7 cells in which DOCK11 was knocked out using the CRISPR-Cas9 system. |
| HepAD38 cells | Obtained from the American Type Culture Collection (Manassas, VA, USA). |
| HepG2 cells | Obtained from the American Type Culture Collection (Manassas, VA, USA). |
| HepG2-NTCP-C4 GHD3 cells | Described in Li YY, et al., Cell Mol Gastroenterol Hepatol 2023 (1). |

Supplemental Table 2. Antibodies used for western blotting and immunofluorescence staining.

| Category | Antibody | Company | Catalogue numeber |
| --- | --- | --- | --- |
| Western blotting | Anti-HaloTag® Monoclonal Antibody | Promega | G9211 |
| Western blotting | Anti-Histone H3 Antibody | Abcam | ab1791 |
| Western blotting | β-Actin Abtibody | Cell Signaling | 4967 |
| Western blotting | PARP1 Antibody | Santa Cruz | sc-8007 |
| Western blotting | DOCK11 Polyclonal Antibody | Bethyl Laboratories | A301-638A |
| Western blotting | Histone H2A.X Antibody | Cell Signaling | 2595 |
| Western blotting | P-Histone H2A.X Antibody | Cell Signaling | 9718 |
| Western blotting | Anti-rabbit IgG HPR-linked Antibody | Cell Signaling | 7074 |
| Western blotting | Anti-mouse IgG HPR-linked Antibody | Cell Signaling | 7076 |
| Immunostaining | Alexa Fluor® 488-conjugated polyclonal DOCK11 antibody | GeneTex | GTX55982 |
| Immunostaining | CoraLite® Plus 647-conjugated PARP1 polyclonal antibody | Proteintech | CL647-13371 |
| Immunostaining | Histone H2A.X Antibody | Cell Signaling | 2595 |
| Immunostaining | PARP1 Antibody | Santa Cruz | sc-8007 |
| Immunostaining | Alexa FluorTM 488 donkey anti-rabbit IgG(H+L) | Invitrogen | A11005 |
| Immunostaining | Alexa FluorTM 594 goat anti-mouse IgG(H+L) | Invitrogen | A21206 |

Supplemental Table 3. Primer sets and a probe for HBV DNA.

| Target - HBV DNA | |
| --- | --- |
| Primer set | 5′-ACTCACCAACCTCCTGTCCT-3′ |
| Primer set | 5′-GACAAACGGGCAACATACCT-3′ |
| Probe | 5′-FAM-TATCGCTGGATGTGTCTGCGGCGT-TAMRA-3′ |

Supplemental Table 4. siRNAs and a PARP inhibitor.

| Target | Type | Company | Catalogue number |
| --- | --- | --- | --- |
| Negative Control | siRNA | QIAGEN | 1027310 |
| PARP1 | siRNA | Thermo Fisher | s1097 |
| PARP1/2 | inhibitor | BPS Bioscience | 27003 |

Supplemental Table 5. 68 proteins determined by LC-MS/MS.

| Gene symbol | Description | Protein FDR Confidence | Peptides | Abundance Ratio  cccDNA formation  (-) | Abundance Ratio  cccDNA formation (+) |
| --- | --- | --- | --- | --- | --- |
| PRKDC | DNA-dependent protein kinase catalytic subunit | high | 213 | 3.106 | 2.872 |
| PARP1 | Poly [ADP-ribose] polymerase 1 | high | 64 | 4.165 | 3.714 |
| XRCC5 | X-ray repair cross-complementing protein 5 | high | 42 | 4.083 | 3.275 |
| XRCC6 | X-ray repair cross-complementing protein 6 | high | 40 | 5.676 | 3.974 |
| UBTF | Upstream binding transcription factor | high | 36 | 4.776 | 17.414 |
| RPA1 | Replication protein A 70 kDa DNA-binding subunit | high | 21 | 5.322 | 5.256 |
| PTBP1 | Polypyrimidine tract-binding protein 1 | high | 21 | 2.196 | 3.763 |
| HIST1H4J | Histone H4 | high | 15 | 5.381 | 4.025 |
| CTCF | Transcriptional repressor CTCF | high | 15 | 2.243 | 3.629 |
| RBM12B | RNA-binding protein 12B | high | 14 | 2.925 | 3.077 |
| TKT | Transketolase | high | 13 | 2.579 | 4.019 |
| GAPDH | Glyceraldehyde-3-phosphate dehydrogenase | high | 12 | 9.976 | 10.316 |
| ATAD2 | ATPase family AAA domain-containing protein 2 | high | 11 | 3.595 | 3.146 |
| MACROH2A1 | Core histone macro-H2A.1 | high | 10 | 8.339 | 4.15 |
| TFCP2 | Transcription factor CP2 | high | 10 | 2.104 | 2.588 |
| PAXX | Protein PAXX | high | 10 | 3.129 | 2.163 |
| TMPO | Lamina-associated polypeptide 2 | high | 9 | 2.055 | 2.943 |
| FKBP3 | Peptidyl-prolyl cis-trans isomerase FKBP3 | high | 9 | 2.17 | 2.715 |
| ZNF362 | Zinc finger protein 362 | high | 8 | 2.596 | 5.255 |
| HMGB1 | High-mobility group box 1 | high | 7 | 3.338 | 5.085 |
| H2BC11 | Histone H2B type 1-J | high | 7 | 9.879 | 3.353 |
| PARP2 | Poly [ADP-ribose] polymerase 2 | high | 7 | 6.89 | 2.231 |
| RPL35 | 60S ribosomal protein L35 | high | 7 | 4.409 | 2.201 |
| AHCY | Adenosylhomocysteinase | high | 6 | 2.816 | 24.149 |
| GIT2 | ARF GTPase-activating protein GIT2 | high | 6 | 2.716 | 5.565 |
| CUL4A | Cullin-4A | high | 5 | 100 | 100 |
| ASS | Argininosuccinate synthase | high | 5 | 9.908 | 100 |
| RAI1 | Retinoic acid-induced protein 1 | high | 5 | 3.314 | 100 |
| RPL36 | 60S ribosomal protein L36 | high | 5 | 3.373 | 8.441 |
| RPA3 | Replication protein A 14 kDa subunit | high | 5 | 3.168 | 4.911 |
| ZC3H7B | Zinc finger CCCH domain-containing protein 7B | high | 5 | 2.256 | 4.854 |
| CENPB | Major centromere autoantigen B | high | 5 | 100 | 2.661 |
| MAPK1 | Mitogen-activated protein kinase | high | 4 | 100 | 100 |
| H2AZ1 | Histone H2A.Z | high | 4 | 10.232 | 7.393 |
| PDCD7 | Programmed cell death protein 7 | high | 4 | 6.041 | 5.682 |
| NQO1 | NAD(P)H dehydrogenase [quinone] 1 | high | 4 | 2.125 | 4.533 |
| RPS29 | 40S ribosomal protein S29 | high | 4 | 7.306 | 4.157 |
| PURA | Transcriptional activator protein Pur-alpha | high | 4 | 2.298 | 4.03 |
| UBA52 | Ubiquitin-60S ribosomal protein L40 | high | 4 | 4.169 | 3.032 |
| PARP14 | Protein mono-ADP-ribosyltransferase PARP14 | high | 4 | 100 | 2.711 |
| JADE1 | Protein Jade-1 | high | 4 | 2.408 | 2.515 |
| DDI2 | Protein DDI1 homolog 2 | high | 4 | 2.139 | 2.491 |
| BRPF3 | Bromodomain and PHD finger-containing protein 3 | high | 4 | 100 | 2.286 |
| NSA2 | Ribosome biogenesis protein NSA2 homolog | high | 4 | 2.345 | 2.073 |
| WASL | Actin nucleation-promoting factor WASL | high | 3 | 100 | 100 |
| ALDH1B1 | Aldehyde dehydrogenase X | high | 3 | 100 | 100 |
| EZH2 | Histone-lysine N-methyltransferase EZH2 | high | 3 | 100 | 100 |
| DECR1 | 2,4-dienoyl CoA reductase 1 | high | 3 | 7.787 | 10.235 |
| AFG2B | Ribosome biogenesis protein SPATA5L1 | high | 3 | 100 | 5.45 |
| REPIN1 | HCG1990708 | high | 3 | 4.919 | 3.709 |
| UHRF2 | E3 ubiquitin-protein ligase UHRF2 | high | 3 | 2.134 | 2.821 |
| NAP1L1 | Nucleosome assembly protein 1-like 1 | high | 3 | 2.299 | 2.529 |
| AJUBA | LIM domain-containing protein ajuba | high | 3 | 2.755 | 2.178 |
| FOXA1 | Hepatocyte nuclear factor 3-alpha | high | 3 | 2.349 | 2.135 |
| UQCRC2 | Cytochrome b-c1 complex subunit 2 | high | 2 | 100 | 100 |
| COMMD9 | COMM domain-containing protein 9 | high | 2 | 100 | 100 |
| ZSCAN2 | Zinc finger and SCAN domain-containing protein 2 | high | 2 | 100 | 100 |
| SMYD3 | Histone-lysine N-methyltransferase SMYD3 | high | 2 | 100 | 100 |
| MCM4 | DNA replication licensing factor MCM4 | high | 2 | 100 | 100 |
| GZF1 | GDNF-inducible zinc finger protein 1 | high | 2 | 100 | 100 |
| GTF2E1 | General transcription factor IIE subunit 1 | high | 2 | 100 | 100 |
| CDK6 | Cyclin-dependent kinase 6 | high | 2 | 100 | 100 |
| TUT4 | RNA uridylyltransferase | high | 2 | 100 | 100 |
| EMSY | BRCA2-interacting transcriptional repressor EMSY | high | 2 | 100 | 100 |
| UBE2E3 | Ubiquitin-conjugating enzyme E2 E3 | high | 2 | 100 | 100 |
| SHOC2 | Leucine-rich repeat protein SHOC-2 | high | 2 | 100 | 5.692 |
| TRIP10 | Cdc42-interacting protein 4 | high | 2 | 4.505 | 3.423 |
| OLA1 | Obg-like ATPase 1 | high | 2 | 2.063 | 2.477 |

Reference

1. Li YY, Kuroki K, Shimakami T, Murai K, Kawaguchi K, Shirasaki T, Nio K, Sugimoto S, Nishikawa T, Okada H, Orita N, Takayama H, Wang Y, Doan PTB, Ishida A, Iwabuchi S, Hashimoto S, Shimaoka T, Tabata N, Watanabe-Takahashi M, Nishikawa K, Yanagawa H, Seiki M, Matsushima K, Yamashita T, Kaneko S, Honda M. 2023. Hepatitis B virus utilizes a retrograde trafficking route via the trans-Golgi network to avoid lysosomal degradation. Cell Mol Gastroenterol Hepatol 15(3):533-558.
